# Supplementary material for: Advancements in Mass Rearing the Air Potato Beetle Lilioceris cheni
Source: Insects. 2022 Jan 6;13(1):65. doi: 10.3390/insects13010065 (PMC8781818; doi:10.3390/insects13010065)
Supplement: Supplementary file 1 [file insects-13-00065-s001.zip › insects-1385467-supplementary.pdf]

Supplemental Materials

Supplementary material S1. Quality Control

In order to ensure our laboratory colony was healthy and retained traits of the introduced beetles we performed a small-scale experiment to test longevity. Twenty-five freshly emerged beetles of both the Nepalese and Chinese biotypes were placed into plastic terrariums with three terrariums of each biotype. Terrariums were purchased at a reptile store and are 28.5 cm × 16.5 cm × 17 cm. Autoclaved vermiculite was used as a substrate. Dried leaf material was placed in terrariums for cover. Fresh leaves were provided to beetles daily and bulbils were also available in each terrarium. All replicates were kept at 27 ± 2 °C, 65 ± 10% RH, and 14L:10D. Water was provided to all treatments via wet cotton wicks. Mortality was recorded daily.

Several beetles escaped over the 14-month course of the experiment, leaving a total of 65 Nepalese and 66 Chinese. Thus, results are presented as percentages. The Scheirer-Ray-Hare test was performed as the data did not pass the test for normality (Shapiro-Wilk W=.86 $p < 0.01$). This analysis shows no significant effect of "biotype" on percent mortality over time (H = 0.62 $p = 0.43$), and a significant effect of "time" was noted (H = 74.97 $p = 0$). There was no significant interaction between "biotype and time" (H = 1.16 $p = 0.10$). Approximately 30% percent of beetles of each biotype died after six months (September). Approximately 90% percent of beetles in each biotype were dead after 11 months (February). Between six to 11 months the death rate of Nepalese beetles was more rapid than Chinese beetles with a range of 56–92 percent mortality of Nepalese biotype and a range of 36–73 percent mortality of the Chinese biotype (Figure S1). Due to this interesting difference a two-way ANOVA was performed ignoring the assumption of normality. This resulted in a significant effect of "biotype", "time", and the interaction of "biotype and time" on percent mortality (F = 10.19 $p < 0.01$; F = 75.30 $p < 0.01$; F = 2.34 $p < 0.01$).

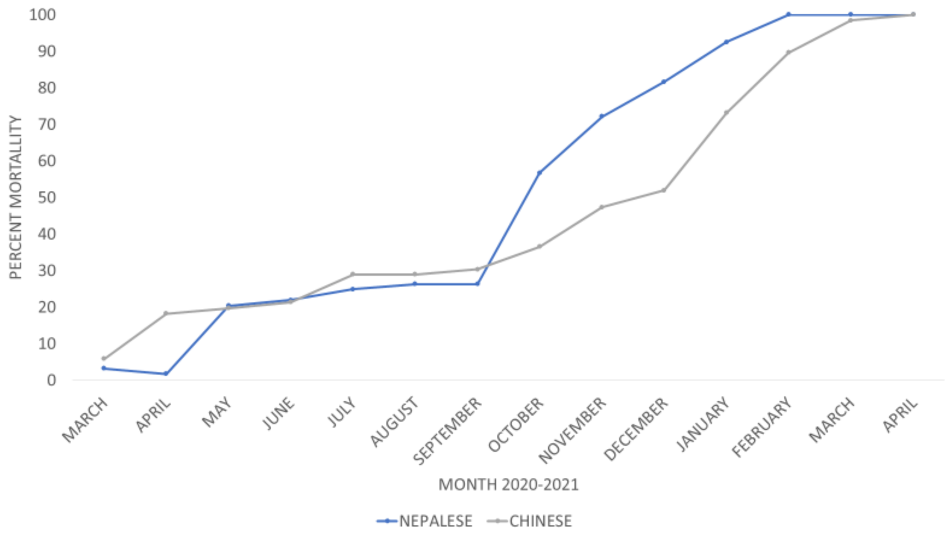

Figure S1. Percent mortality of the two air potato beetle biotypes over a 14-month period.

Supplementary material S2. Equipment and supply list

- Field Cages (Figure S2-1)
- Humidifiers
- Dehumidifier
- RO (Reverse Osmosis) water system
- Pesticide sprayer
- Hose with spray-nozzle attachment
- Automatic timer for lights
- T5VHO lighting fixtures (48 in.)
- Logbooks or data logger
- Nursery containers (10 gallon) (Figure S2-2)
- Potting mix
- Slow-release complete fertilizer
- Micronutrient fertilizer
- Bone meal
- Horticultural oil
- Insecticidal soap

- Pyrethrum micro-fogger
- 6′ & 8′ bamboo stakes (Figure S2-3)
- Garden clippers
- Tray or cardboard box
- Container for bulbils
- Latex/nitrile gloves
- 4L storage tubs with screened ventilation holes in lid (Figure S2-4)
- 12L clear storage bins
- Self-adhesive label tape
- Medium-grade vermiculite
- Square net with extendable pole
- Tri-fold paper towels
- Plastic spray bottles
- Dish soap
- 70% isopropyl alcohol
- Moistened wicks (Figure S2-5)

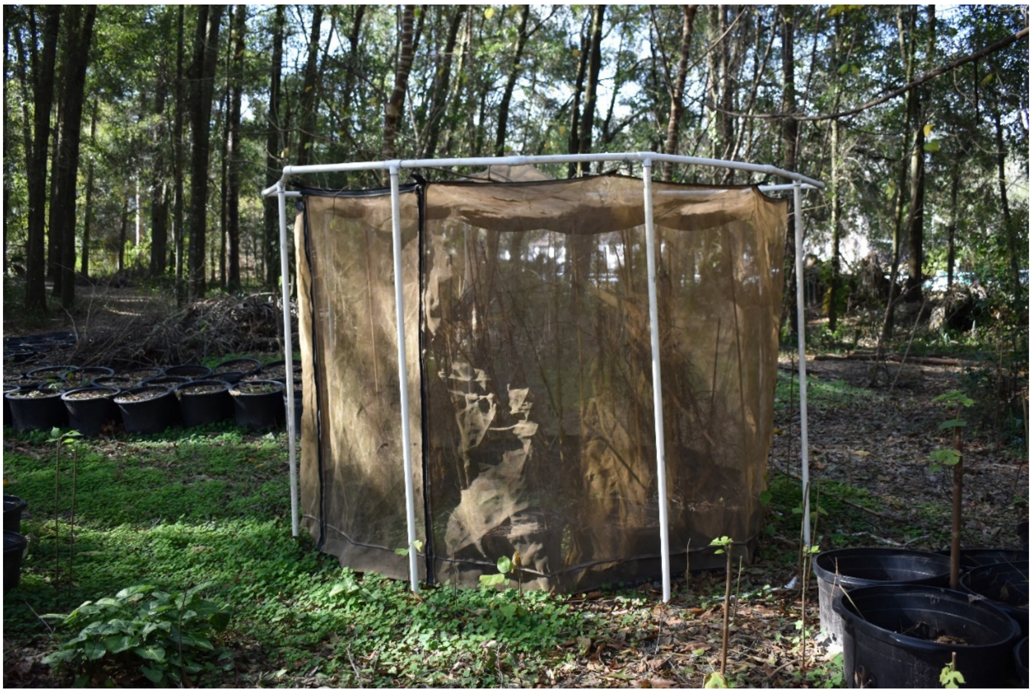

Figure S2-1: Field cage.

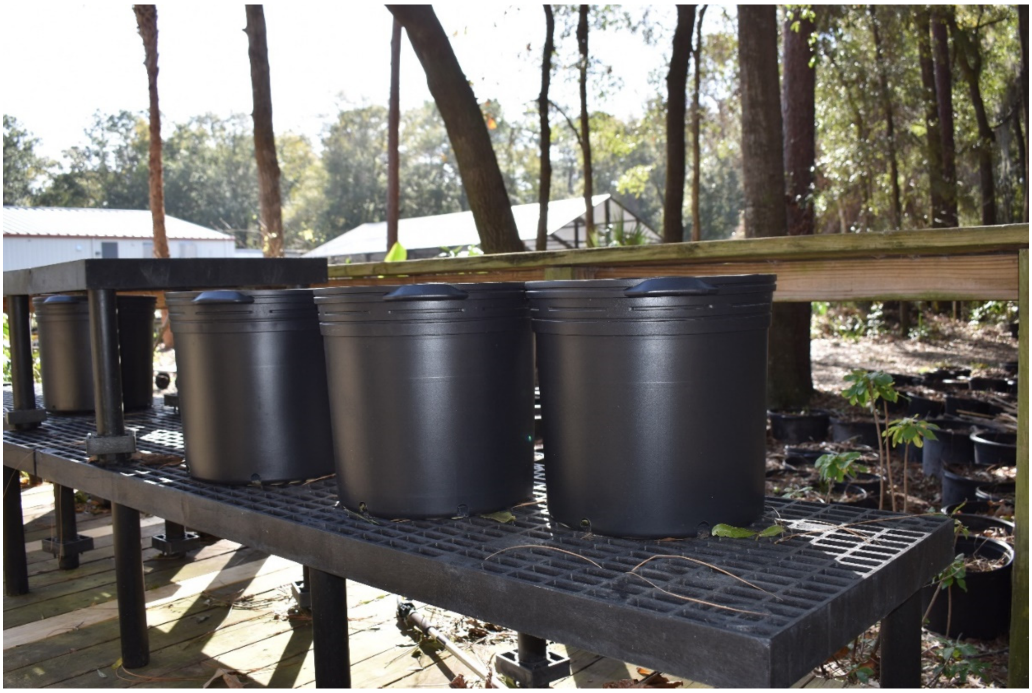

Figure S2-2: 10-gallon container.

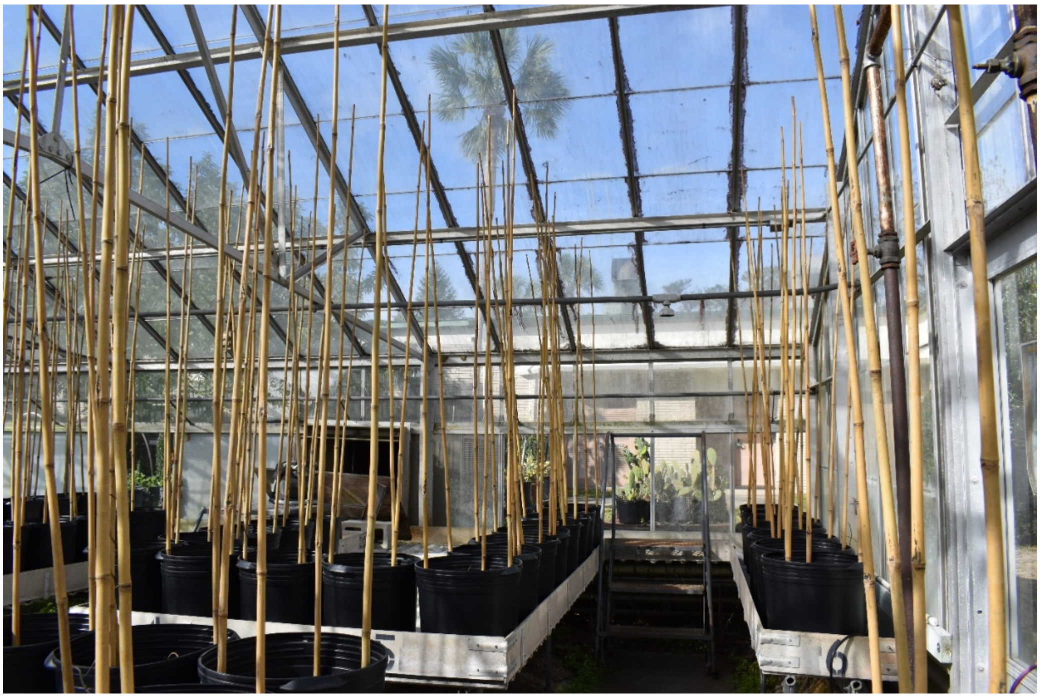

Figure S2-3: Bamboo poles in 10-gallon containers.

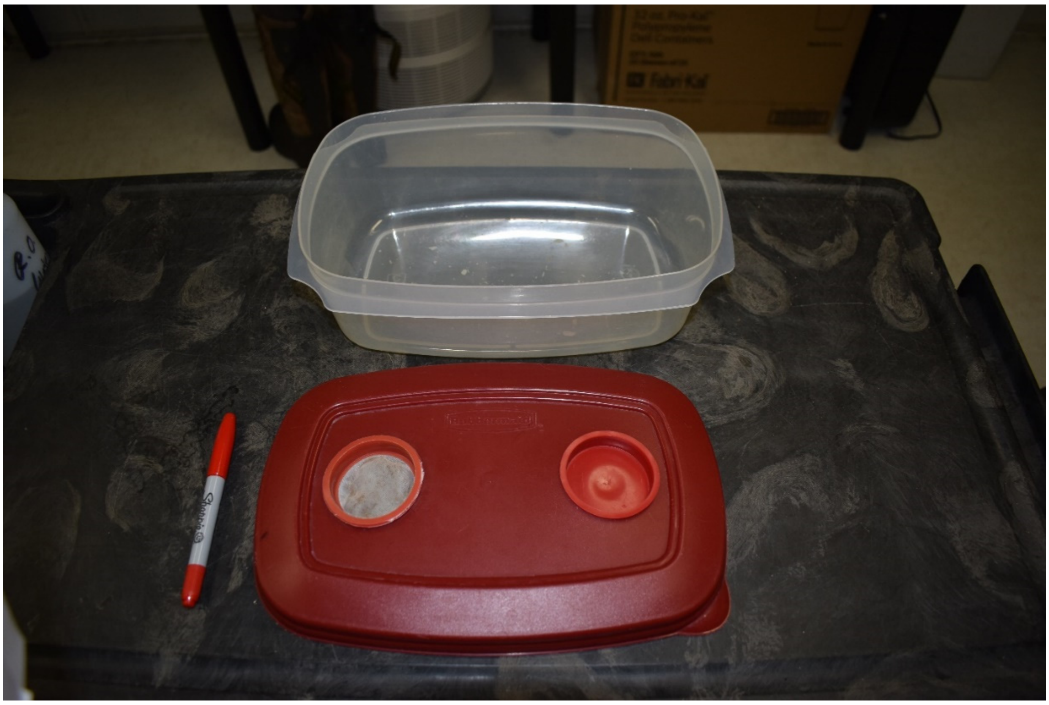

Figure S2-4: 4-Liter storage tub with screened ventilation in lid.

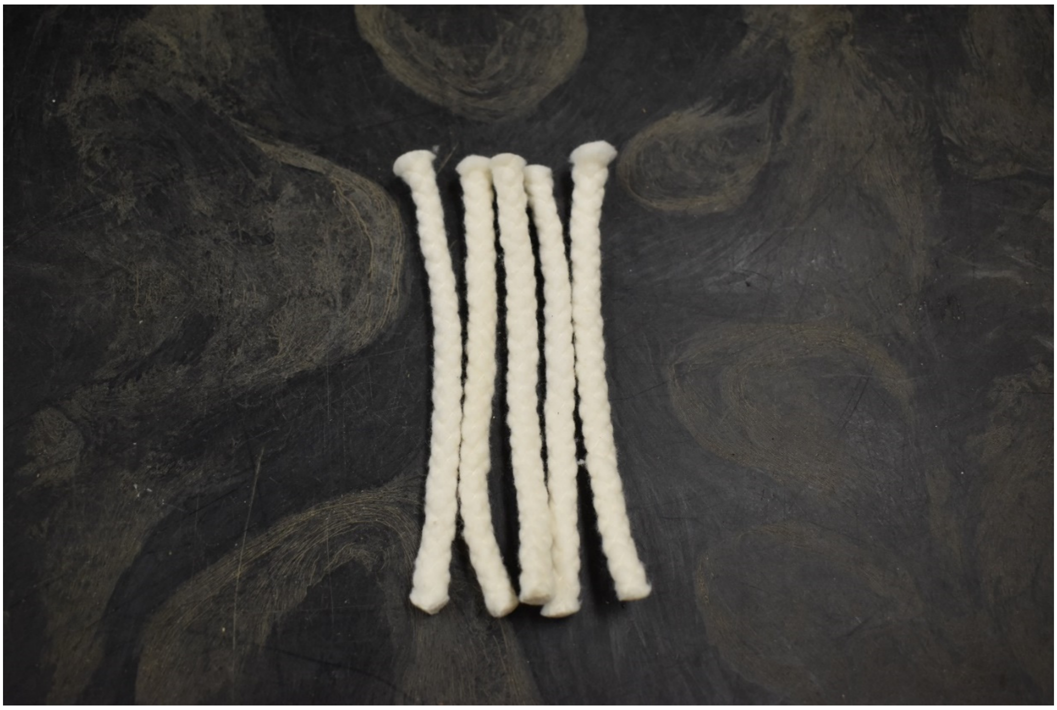

Figure S2-5: Cotton wicks.

Supplementary material S3. Example of instructions to send with beetle shipments.

**When you are finished distributing your beetles, please use the USPS return label in the box to return the package and packing contents. It is pre-paid; all you need to do is tape it somewhere on the box (or put it in the plastic mailer envelope) and send it back. * (Note: you do NOT need to fill out your address on the return label)*

Release Instructions: Do not separate the beetles out of their deli cups, KEEP THEM ALL TOGETHER

To release the beetles simply empty out the plastic container of beetles and air potato leaves near your air potato vine. You do not need to place them on the vine, they will disperse themselves. We recommend releasing all the beetles in one spot so that they can mate, increase their population size and thus increase the damage done to the air potato vines.

Please note that the air potato beetles will disperse themselves, as they can fly miles from their release point!

Be patient, it takes some time for the beetles to become effective

Please keep in mind it will take a few weeks for your beetles to get settled in. Once settled, they will lay eggs which will hatch in 2–4 days, then the larvae (babies) will feed for about a week. When the larvae are ready to pupate (metamorphose), they will move to the soil. In about two weeks they will emerge from the soil as adult beetles. About a week after emergence the adult

beetles will start laying eggs and the cycle will continue once again. *The entire life cycle from egg to adult takes about a month and the beetles live on average 6 months.*

Adult beetle damage will be noticed first, then larval damage. Once the larvae begin eating you should start seeing a *drastic* increase in damage to the air potato vines.

The air potato beetles will usually come back each year

When the air potato vines senesce around October the air potato beetles will enter diapause in order to overwinter. During diapause, they will sleep for a long period of time in the leaf litter, until they re-emerge in the spring, about a month after the air potato vines start growing again. Re-emergence is variable and is based on fall/winter's weather conditions.

But, they will not eradicate your vine

While the beetles will feed on older leaves and occasionally the bulbils (air potatoes), they prefer to eat the new growth. This hinders its growth significantly, prevents it from climbing high into the canopy and prevents the vine from choking other plants. Most importantly, the damage the beetles cause reduces the vine's resources, meaning a reduction in the number of new bulbils (air potatoes) produced and thus limits the spread of the vine. If you are interested in eradicating air potato vine from your yard, it is recommended you also manually remove the vine's means of propagation and energy storage—the aerial bulbils and underground tubers.

Best wishes with your release, and if you have any questions or concerns please do not hesitate to reach out to me!
